# *Aedes albopictus* and *Aedes japonicus* - two invasive mosquito species with different temperature niches in Europe

**DOI:** 10.1186/s13071-016-1853-2

**Published:** 2016-11-04

**Authors:** Sarah Cunze, Lisa K. Koch, Judith Kochmann, Sven Klimpel

**Affiliations:** 1 Institute of Ecology, Evolution and Diversity, Goethe-University, D-60438 Frankfurt/ M., Germany; 2 Senckenberg Biodiversity and Climate Research Centre, Senckenberg Gesellschaft für Naturforschung, D-60438 Frankfurt/ M., Germany

**Keywords:** Asian bush mosquito, Asian tiger mosquito, Climate change, Invasive species, Species distribution modelling

## Abstract

**Background:**

*Aedes albopictus* and *Ae. japonicus* are two of the most widespread invasive mosquito species that have recently become established in western Europe. Both species are associated with the transmission of a number of serious diseases and are projected to continue their spread in Europe.

**Methods:**

In the present study, we modelled the habitat suitability for both species under current and future climatic conditions by means of an Ensemble forecasting approach. We additionally compared the modelled MAXENT niches of *Ae. albopictus* and *Ae. japonicus* regarding temperature and precipitation requirements.

**Results:**

Both species were modelled to find suitable habitat conditions in distinct areas within Europe: *Ae. albopictus* within the Mediterranean regions in southern Europe, *Ae. japonicus* within the more temperate regions of central Europe. Only in few regions, suitable habitat conditions were projected to overlap for both species. Whereas *Ae. albopictus* is projected to be generally promoted by climate change in Europe, the area modelled to be climatically suitable for *Ae. japonicus* is projected to decrease under climate change. This projection of range reduction under climate change relies on the assumption that *Ae. japonicus* is not able to adapt to warmer climatic conditions. The modelled MAXENT temperature niches of *Ae. japonicus* were found to be narrower with an optimum at lower temperatures compared to the niches of *Ae. albopictus.*

**Conclusions:**

Species distribution models identifying areas with high habitat suitability can help improving monitoring programmes for invasive species currently in place. However, as mosquito species are known to be able to adapt to new environmental conditions within the invasion range quickly, niche evolution of invasive mosquito species should be closely followed upon in future studies.

**Electronic supplementary material:**

The online version of this article (doi:10.1186/s13071-016-1853-2) contains supplementary material, which is available to authorized users.

## Background

Climate change is assumed to generally promote the invasive success of introduced species. In addition to finding more suitable conditions, they can indirectly benefit from changed climatic conditions as some ecosystems might become less resistant to invasion [1]. Species distribution models are a useful and commonly applied tool to project climate change induced range shifts of species (e.g. [2]). Species distribution models can improve assessments of species’ invasive potential and guide management actions [3]. This is especially important for species that function as vectors, which may potentially pose a threat to human health.

Ecological niche modelling is a commonly used correlative approach to model the habitat suitability for a species under current and projected future climatic conditions. Based on a species’ presence/absence information, and environmental conditions at a particular geographical location, species-habitat relationships are estimated [4] by means of different statistical algorithms, providing data on relative habitat suitability. Based on the information where the species is present (and in addition ideally where the species is absent) and environmental conditions prevail there, the species-habitat-relationship is estimated [4] by means of different statistical algorithms. This modelled species-habitat relationship (niche function) can then be projected onto the study area, resulting in a habitat suitability map for the considered species within the considered area. This projection can be based on the data on current climatic conditions, thus reflecting the potential distribution under current climatic conditions. In addition, data on potential future climatic conditions, like those provided by the Intergovernmental Panel on Climate Change (IPCC), can be used, which would represent the potential future distribution of the considered species.

Projections on habitat suitability under future climatic conditions may help to assess the invasive potential of non-native species. Climate warming is assumed to yield a north eastwards shift and a shift to higher altitudes of areas with suitable habitat conditions for many species. Projections on habitat suitability for invasive species under future climatic conditions may help improving existing monitoring programmes as well as preventing negative consequences for ecosystems and human health in the future.

*Aedes albopictus* and *Ae. japonicus* are two of the most widespread invasive mosquito species worldwide. Native to Asia, these two species have been spreading rapidly across the globe with increasing transport of certain goods and facilitated by human activities such as travelling [5, 6]. Whereas *Ae. albopictus* originates in the forests of tropical regions of south-east Asia [5–7], *Ae. japonicus* has been originally restricted to Japan, north of the Ryukyu Islands (Hokkaido, Honshu, Shikoku, and Kyushu) and the Korean Peninsula, where it is common, even in large cities, but not particularly abundant [8]. *Aedes japonicus* does not occur in the tropics, but both species co-occur naturally in Japan and Korea [9].

*Aedes albopictus* is considered to be one of the world’s fastest-spreading invasive animal species [10]. *Aedes japonicus* has spread throughout North America and later into central Europe at a rate comparable to that of *Ae. albopictus* [8]. The rapid global spread of these species is certainly favoured by extrinsic factors such as globalization and climate change [11]. International as well as intercontinental trade (e.g. of used tyres that may act as breeding places) may have facilitated the introduction of these species [12]. Furthermore, the successful invasion of *Ae. albopictus* is assumed to be promoted by intrinsic factors (i.e. factors that act from within the species) such as strong ecological plasticity, which allows the species to get successfully established in a wide range of different habitats with different climatic conditions [12]. Both species produce desiccation-resistant eggs. This trait likely facilitated the transport and consequently, the successful introduction of *Ae. albopictus* and *Ae. japonicus* to many places worldwide [9, 13]. In addition, the eggs of both species can undergo diapause and thus overwinter in temperate climates despite the adult forms being unable to survive through this period (e.g. [14–16]).

*Aedes albopictus* as well as *Ae. japonicus* are known to function as competent vectors for a number of serious diseases [17, 18] including dengue fever, yellow fever, West Nile fever and Rift Valley fever [6] as well as Japanese encephalitis [19]. Due to this public health significance, the interest in investigating the establishment and spread of invasive mosquitoes in Europe has been on the rise for the past few years. Research including monitoring the current spread of these species, modelling the potential future spread and also studies on the species’ ecology is considered to be particularly important in order to contain their further spread [6].

In the present study, we focused on the spread of *Ae. albopictus* and *Ae. japonicus* in Europe. Both species are considered invasive in many countries worldwide, including North America and Africa. More recently, they have also become established in western Europe [13, 20, 21]. Due to ongoing introductions and projected climate change, both species are predicted to continue their spread in Europe and will therefore remain the subject of surveillance and monitoring programmes [22]. As a species adapted to warmer climatic conditions, *Ae. albopictus* is assumed to be strongly promoted by projected climate change (e.g. [17, 23]. Compared to *Ae. albopictus, Ae. japonicus* is assumed to be adapted to colder temperatures. Thus, one may hypothesize that *Ae. japonicus* will not particularly benefit from projected long-term global warming (or to a lesser extent) compared to *Ae. albopictus*. However, despite or even because of being able to withstand winter temperatures, and due to the recorded rapid spread in Switzerland, *Ae. japonicus* is projected to become more widely established in Europe in the following years [24].

We here used correlative species distribution modelling, based on ten commonly applied niche modelling algorithms (Ensemble forecasting), to project and compare the modelled habitat suitability for *Ae. albopictus* and *Ae. japonicus* in Europe under current and future climatic conditions. The first step comprised modelling the habitat suitability for the two species in Europe. Due to the assumed different climatic requirements, we expected the species to find suitable habitat conditions in different regions of Europe. We hypothesized that *Ae. albopictus* would clearly benefit from climate change resulting in an enlargement of the area with modelled suitable habitat conditions, but that *Ae. japonicus* would do so only to a lesser extent. As environmental variables we considered six variables covering temperature and precipitation conditions (mean temperature of coldest quarter, mean temperature of warmest quarter, temperature annual range, annual mean precipitation, annual precipitation as well as precipitation in warmest quarter).

To derive more specific information on the species’ ecology from the occurrence records in combination with environmental variables (temperature and precipitation), the second step was to compare the modelled MAXENT niches of the two species by means of so-called one-variable response curves. We expected that *Ae. japonicus* would show a smaller temperature niche with an optimum shifted to colder temperatures compared to *Ae. albopictus*. Considering *Ae. albopictus* and *Ae. japonicus* requirements of small aquatic habitats for breeding, we hypothesized that both species would have similar niches considering precipitation variables with regard to location and amplitude.

## Methods

### Occurrence data

Occurrence data are taken from Kraemer MUG et al. [25] and Koch et al. [17] for *Ae. albopictus* and from Schaffner et al. [26]; Huber et al. [27]; Huber et al. [19]; Krebs et al. [28]; Zielke et al. [29]; Melaun et al. [18] and Zielke et al. [30] for *Ae. japonicus*. The original coordinates were adjusted to the raster of the environmental variables (about 10 × 10 km).

The records were adjusted to the raster of the environmental variables. The records for *Ae. albopictus* date back to the period between 1980 and 2015 and the records for *Ae. japonicus* date back to the period between 2000 and 2015, and cover the area of the currently known distribution of both species. We modelled the habitat suitability for both species in Europe considering the following spatial extent: Latitude, 35°N-79°N; Longitude, 10°W-45°E; and worked at a spatial resolution of five arc min (~10 km). The maps were built using ESRI ArcGIS (Release 10.3, www.esri.com).

### Environmental data (current and future climatic conditions)

Here, six climatic variables were taken into account, including mean temperature of coldest quarter, mean temperature of warmest quarter, temperature annual range (maximal temperature of warmest month - minimal temperature of coldest month), annual mean precipitation, precipitation in warmest quarter and precipitation seasonality (coefficient of variation). We thus accounted for temperature conditions during summer and winter, summer precipitation and annual precipitation as well as the variation of these two factors during the year. We decided to use annual precipitation instead of winter precipitation as the latter is assumed to be ecologically irrelevant for both species. This selection of variables from the 19 bioclimatic variables available from worldclim [31] (www.worldclim.org/) was based on a correlation analysis coupled with assumptions according the ecological relevance of the variables for the species. The considered variables are not strongly inter-correlated (Spearman correlation coefficient < 0.7, see Additional file 1: Table S1). Temperature in winter is regarded as critical for the survival of individuals (of different stages), summer temperature is assumed to influence the activity of individuals, temperature range may be important determining the east-west gradient of species in Europe, precipitation is considered crucial as both mosquito species require small aquatic habitats for breeding, whose general availability is assumed to be connected to precipitation conditions. Data on current climatic conditions were provided by worldclim. Data on future climatic conditions based on the fifth IPCC Assessment report (AR5) were taken from the International Centre for Tropical Agriculture (CIAT) and The CGIAR Research Program on Climate Change, Agriculture and Food Security (CCAFS) [32] (http://ccafs-climate.org/data/).

The IPCC is a scientific intergovernmental body that produces reports which have the agreement of leading climate scientists and the consensus of participating governments. Based on the IPCC reports a range of emission scenarios are derived for use in global climate models that project future climatic conditions under different socio-economic and emission scenarios. Socio-economic and emission scenarios are considered in climate research to provide plausible descriptions of how the future may evolve with respect to a range of variables including socio-economic change, technological change, energy and land use and emissions of greenhouse gases and air pollutants. They are used as input for climate model runs and as a basis for assessment of possible climate impacts and mitigation options and associated costs. In the IPCC AR5 four so-called ‘Representative Concentration Pathways’ (RCPs) are considered that represent a broad range of climate outcomes. The RCPs describe four possible climate futures, all of which are considered possible depending on how much greenhouse gases are emitted in the years to come.

RCP 2.6 is based on the assumption that the maximum of global annual greenhouse gas emissions takes place between 2010 and 2020 with a substantial decline thereafter. According to the RCP 4.5 and RCP 6.0, global annual greenhouse gas emissions will rise until 2040 and 2080, respectively, and then decline. In RCP 8.5, the global annual greenhouse gas emissions are assumed to rise throughout the 21st century [33].

We projected the habitat suitability for *Ae. albopictus* and *Ae. japonicus* for four time periods, including under current climatic conditions (based on measurements between 1950 and 2000) and under future climatic conditions (for three future time periods from 2021–2040, 2041–2060 and 2061–2080). We considered four different RCPs (5th Assessment report [34]) to account for uncertainty in data on future climatic conditions.

### Ensemble forecasting

Modelling results often show a high variability, which can be ascribed to the sensitivity of correlative models to the data and the mathematical functions used to describe the species-habitat relationship [35]. Comparative studies using independent presence/absence data for evaluation (e.g. [36, 37]) could not verify the superiority of one single algorithm [35] although generating a lot of knowledge regarding modelling performance [38]. One way to deal with the uncertainty of SDM algorithms is the so-called Ensemble forecasting. Ensemble forecasting denotes the application of several alternative statistical algorithms [38]. The results of these different models are then integrated into one ‘consensus’ model, which provides more robust estimates [35]. Different methods exist to create the consensus model (see [38]) and here we chose to use weighted averages.

In order to yield habitat suitability maps for both mosquito species considered here, we run an Ensemble forecasting approach based on ten state-of-the-art algorithms (GLM, generalized linear models; GAM, generalized additive models; GBM, generalized boosted models; CTA, classification tree analysis; SRE, surface range envelope; ANN, artificial neural networks; FDA, flexible discriminant analysis; MARS, multivariate adaptive regression splines; RF, random forest; MAXENT, maximum entropy approach) implemented in the R package BIOMOD2 (version 3.3-7; [39]). For a short description of the used algorithms see [37] and [40].

### Modelling performance and variable importance

The AUC-value (i.e. the area under the receiver operating characteristic curve value) is a threshold independent measure for modelling performance that ranges between 0 (low performance) and 1 (high performance). As a consensus model we considered the mean average of ten algorithms weighted by the AUC value [38] (required an AUC value of the single models of over 0.75). For binary modelling results, we applied the sensitivity equals specificity threshold [41].

Regarding environmental variables relevant for the potential distribution of the two mosquito species we compared the relative contributions of the six environmental variables to the MAXENT models for the two species. In order to calculate the MAXENT permutation variable importance as a measure of relative variable importance the values of each variable in turn are randomly permuted on training presence and background data. The model is re-evaluated on the permuted data, and the resulting drop in training AUC, normalized to percentages is then taken as a measure of relative variable importance.

### MAXENT

In addition to providing habitat suitability maps for the two mosquito species under current and future climatic conditions, we aimed to further investigate the modelled species-habitat relationship with regard to a comparison of the modelled temperature and precipitation niches of the two species. For this, we focused on the maximum entropy niche modelling approach (MAXENT, [42, 43]) for the following reasons: the MAXENT approach is one of the most commonly employed algorithms to model species potential ranges (e.g. [44]), it scores well in comparative studies (e.g. [37]) and the modelled niche function is relatively easy to handle from a mathematically point of view [45]. According to Baldwin [46], the maximum entropy approach is relatively insensitive to spatial errors associated with location data, requires few locations to construct useful models and performs better than other presence only modelling algorithms. These may be especially important issues considering *Ae. albopictus* and *Ae. japonicus* as non-native invasive species in Europe. We used the Maximum entropy approach as implemented in the freeware MAXENT (version 3.3.3 k [42, 43]). We used the default setting with 20 replications but only linear, quadratic and product features (c.f. [45]). One-variable models were displayed as one-variable-response-curves using R (version 3.2.1, www.r-project.org) and compared between the two species. These curves reflect the dependence of predicted habitat suitability on each environmental variable. We only show response curves for variables for those the one-variable-models score an AUC value of at least 0.75 for both species.

## Results

### Ensemble forecasting under current and future climatic conditions

**Fig. 1:**
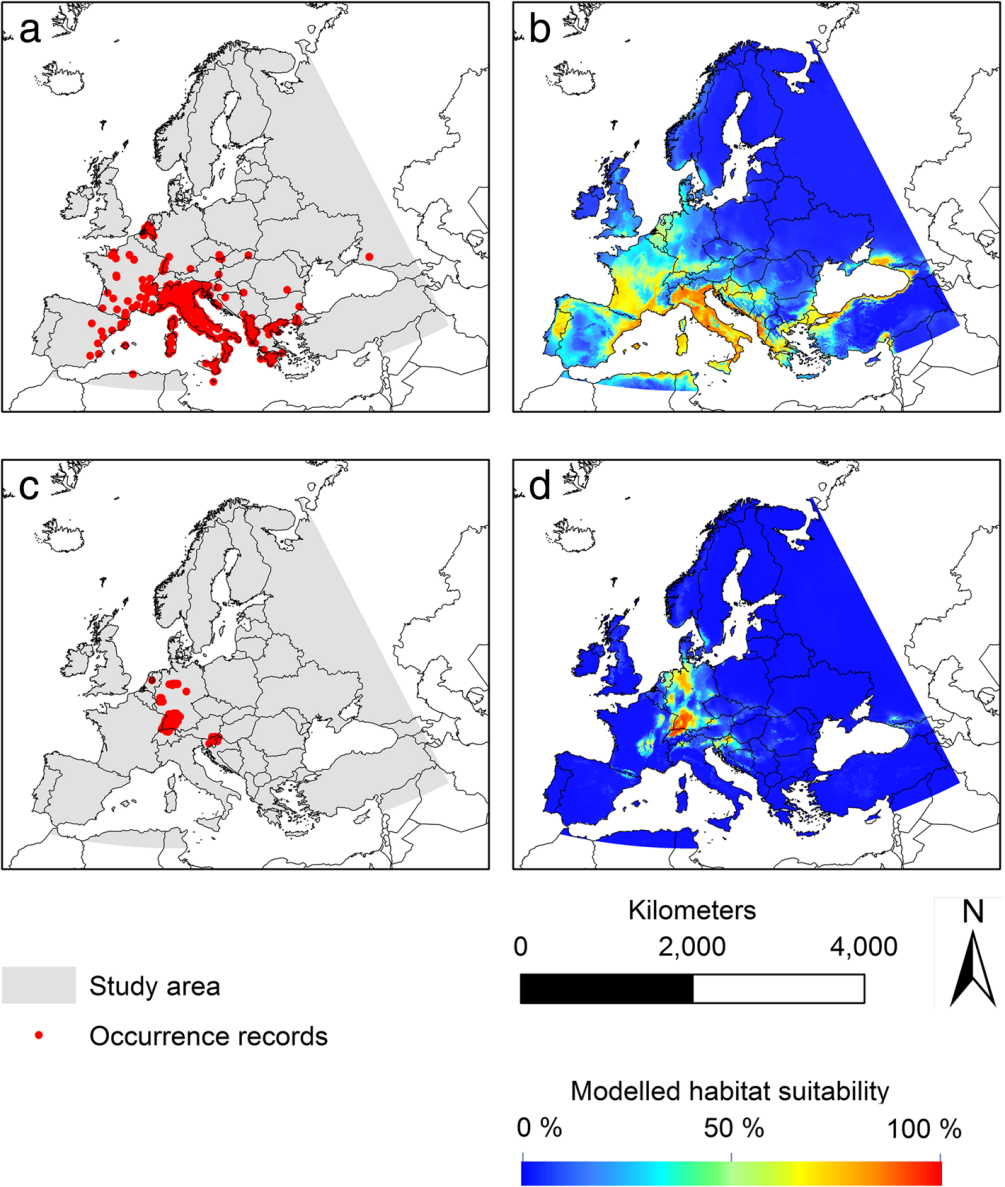
Observed and modelled European distribution for *Aedes albopictus* and *Ae. japonicus.* **a** Occurrence records for *Ae. albopictus* (*n* = 336). **b** Modelled habitat suitability for *Ae. albopictus* under current climatic conditions, Ensemble forecasting (AUC = 0.972). **c** Occurrence records for *Ae. japonicus* (*n* = 178). **d** Modelled habitat suitability for *Ae. japonicus* under current climatic conditions, Ensemble forecasting (AUC = 0.999)

**Fig. 2:**
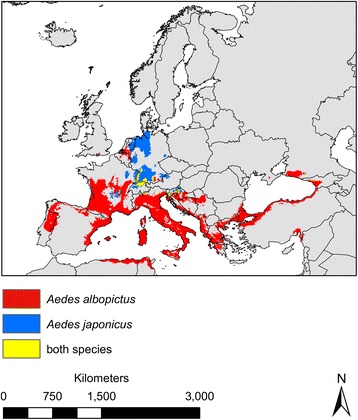
Area with modelled habitat suitability (Ensemble forecasting) in Europe for *Aedes albopictus* and *Aedes japonicus* and both mosquito species under current climatic conditions. Sensitivity equals specificity threshold was applied to yield binary modelling results

According to our modelling results, suitable habitat conditions for the two invasive mosquito species *Ae. albopictus* and *Ae. japonicus* can currently be found in distinct areas within Europe (Figs. 1 and 2). Suitable habitat conditions and thus potential distribution of *Ae. albopictus* was projected to be currently restricted to southern Europe, which can be characterized by Mediterranean climatic conditions (warm or hot, dry summers and mild or cool, wet winters). In contrast, *Ae. japonicus* is projected to find suitable conditions within the climatically more temperate regions in central Europe. Only in a few regions (the Upper Rhine Valley in Germany, France and Switzerland, parts of southern Germany, Switzerland and Slovenia as well as small regions in northern Italy, Austria and Croatia) both species are projected to find currently suitable climatic conditions, potentially leading to a co-occurrence in these regions (Fig. 2).

For both species the modelled habitat suitability (Fig. 1b, d) reflect the observed distribution (Fig. 1a, c) of both species quite well. The AUC values for the consensus models resulting from the Ensemble forecasts are high, with 0.972 for *Ae. albopictus* and 0.999 for *Ae. japonicus*. The AUC values for the single models are given in Additional file 2: Table S2.

**Fig. 3:**
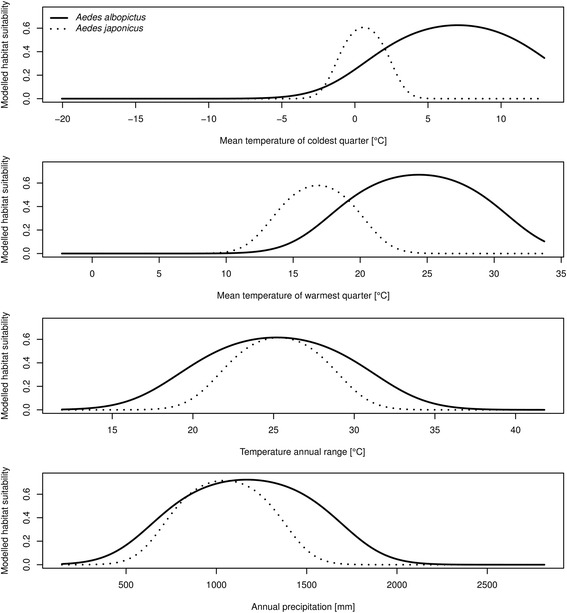
Modelled habitat suitability (Ensemble forecasting) for *Aedes albopictus* under current and future climatic conditions

*Aedes albopictus* is modelled to expand its potential range in Europe north-eastwards under future climatic conditions (Fig. 3), with large parts of France, the Benelux region and parts of Germany becoming climatically suitable for the species. Generally, the climatic suitability will increase area-wide across nearly whole Europe. These rises are consistent over all four RCP-scenarios. Under the RCP 2.6, which is associated to the lowest temperature increase in the face of climate change, the increase of projected habitat suitability for *Ae. albopictus* is comparably lowest, whereas the projected increase of habitat suitability is highest for the RCP 8.5 which is associated to the highest temperature increase.

**Fig. 4:**
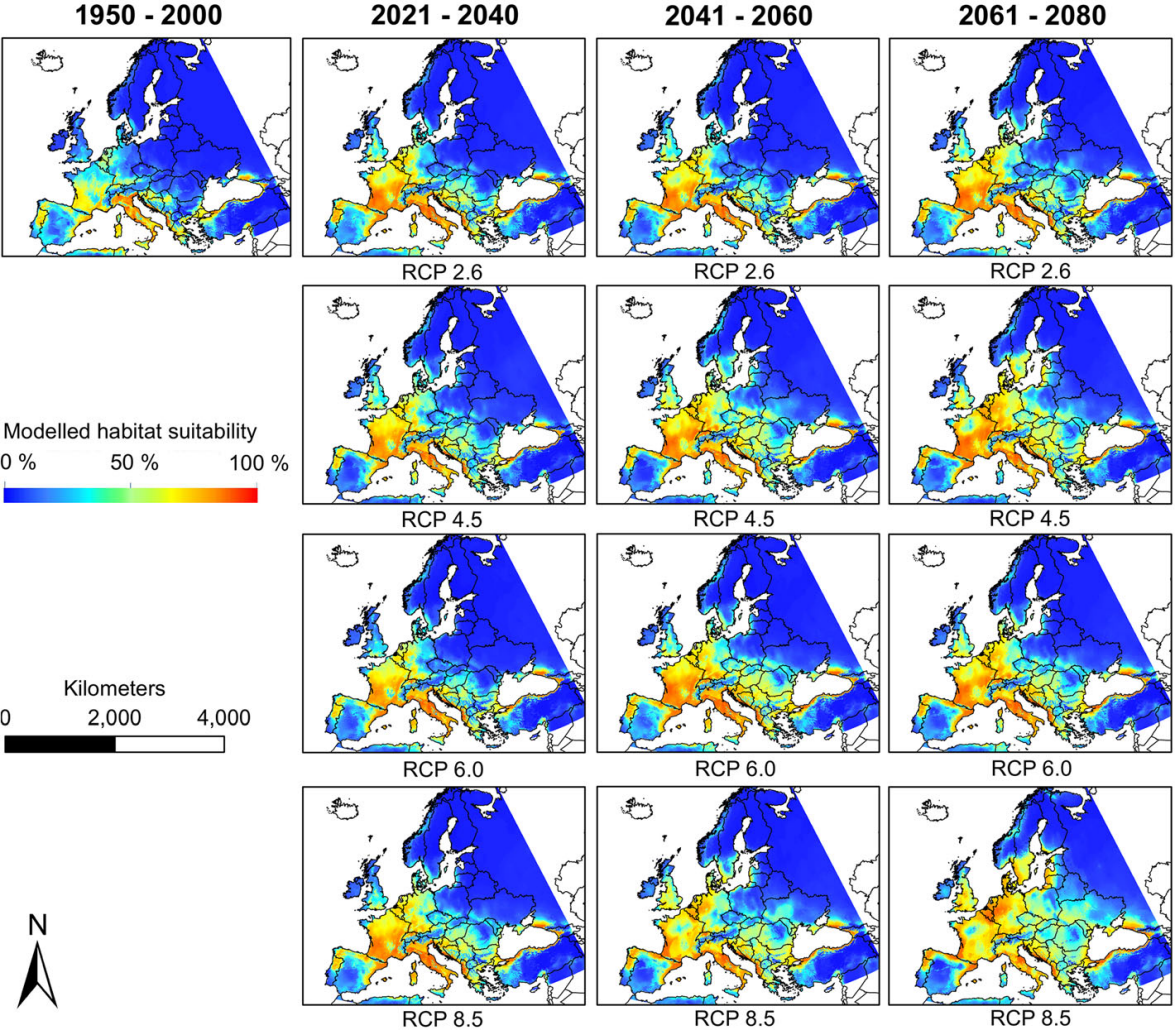
Modelled habitat suitability (Ensemble forecasting) for *Aedes japonicus* under current and future climatic conditions

Compared to the increased habitat suitability for *Ae. albopictus, Ae. japonicus* is projected to find decreasingly suitable habitats under future climatic conditions (Fig. 4). Depending on the time period and RCP considered, *Ae. japonicus* is modelled to only find suitable habitat conditions in southern Germany and very small regions in the High Tatras.

### Variable importance

Considering the permutation variable importance as a measure for the variables contribution to the MAXENT model (Table 1), the mean temperatures of coldest quarter showed for both species the highest values. In second place are mean temperature of warmest quarter for *Ae. albopictus* and annual precipitation for *Ae. japonicus*.

**Table 1:** Relative variable importance (MAXENT) in %. The permutation importance provides estimates of relative contributions of the environmental variables to the MAXENT model

Variables	*Ae. albopictus*	*Ae. japonicus*
Mean temperature of warmest quarter	22.9	1.9
Mean temperature of coldest quarter	50.3	42.2
Temperature annual range	7.3	0.2
Precipitation of warmest quarter	6.6	11
Annual precipitation	9.2	39.5
Precipitation seasonality	3.6	5.2

### One-variable response curves (MAXENT)

**Fig. 5:**
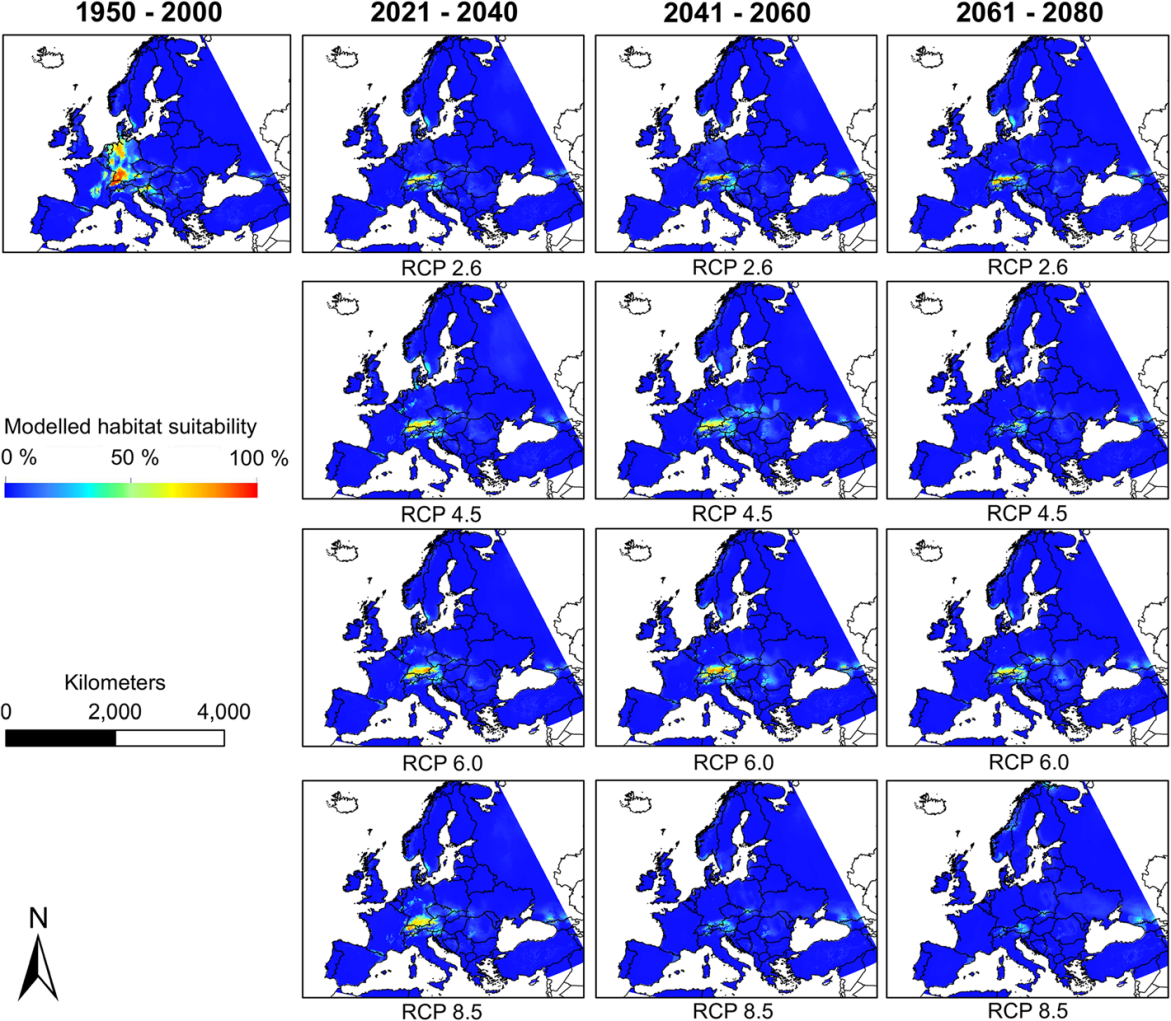
One-variable-response-curves for *Aedes albopictus* (*solid line*) and *Aedes japonicus* (*dotted line*) considering the different environmental variables (required: AUC value for the one variable model for both species > 0.75, see Table 2)

As depicted in the one-variable response curves (Fig. 5), the modelled niche function for *Ae. japonicus* shows a lower temperature optimum than that of *Ae. albopictus*; this is true for the variables mean temperature of the coldest quarter and mean temperature of the warmest quarter. Furthermore, *Ae. japonicus* is modelled to have a narrower niche considering these variables as well as for the variables temperature annual range and annual precipitation. For the latter two variables the modelled optima are rather similar for the both species. The response curves for the variables precipitation of warmest quarter and precipitation seasonality are not shown in Fig. 5 as the one-variable models for at least one of the two considered mosquito species has an AUC value of less than 0.75 and thus, the one-variable model is of low predictive power. The AUC values for the one-variable models for all six variables and the two species are given in Table 2.

**Table 2:** AUC values for the one-variable models, mean average over 20 replications ± standard deviation. Response curves for the variables for which the one variable model yields an AUC value of at least 0.75 for both species are shown in Fig. 5

Variable	*Ae. albopictus*	*Ae. japonicus*
Mean temperature of warmest quarter	0.818 ± 0.002	0.840 ± 0.003
Mean temperature of coldest quarter	0.851 ± 0.001	0.924 ± 0.001
Temperature annual range	0.790 ± 0.001	0.915 ± 0.002
Precipitation of warmest quarter	0.593 ± 0.004	0.901 ± 0.002
Annual precipitation	0.764 ± 0.002	0.864 ± 0.002
Precipitation seasonality	0.554 ± 0.003	0.779 ± 0.002

## Discussion

Mosquitoes can pose a serious threat to human health as they function as vectors for serious diseases. Facing risks from undeliberate introductions of non-native species together with range expansions facilitated by changing temperature regimes, modelling distribution of vector species is urgently needed. Here, we modelled the ecological niches and habitat suitability for the two mosquito species *Ae. albopictus* and *Ae. japonicus* within their invasive range under current and future climate in Europe using an Ensemble forecasting approach based on ten commonly applied niche modelling algorithms.

The results are broadly consistent with previous studies [17, 18, 23] despite different algorithms, different sets of explaining variables and different, more recent sets of occurrence data. This robustness of modelling indicated the reliability of modelling results.

The pattern of areas with modelled suitable habitat conditions as well as the one-variable response curves reflect the differences in temperature requirements of both species. Under current climate, *Ae. albopictus,* assumed to be adapted to warmer temperatures (Additional file 3: Table S3), is projected to find suitable conditions in the Mediterranean region, where generally higher temperatures prevail. *Ae. japonicus,* assumed to be adapted to comparably colder temperatures (Additional file 3: Table S3), is projected to find suitable habitat conditions in the more temperate regions in central Europe. These results are also reflected by a narrower modelled niche for *Ae. japonicus* with lower temperature optima compared to *Ae. albopictus*.

### Potential distribution under current climatic conditions

Both species find suitable habitat conditions under current climate in distinct areas within Europe: *Ae. albopictus* within the Mediterranean regions in southern Europe and *Ae. japonicus* within the more temperate regions of central Europe. Only in a few regions do suitable habitat conditions overlap for both species. These results are in accordance with the currently observed distribution of the species (Additional file 4: Figure S1). The first European record for *Ae. albopictus* dates back to 1979, when the species was found in Albania. In 1990, *Ae. albopictus* was recorded for the first time in Italy. Since then, the species has successfully established in large parts of the Mediterranean region in southern Europe [24] (e.g. Italy [47–49], southern France [50] and Romania [51]). *Aedes japonicus* was recorded for the first time in 2000 in France, but was quickly eradicated [52]. Currently*, Ae. japonicus* is known from six European countries [30], including Belgium [26], The Netherlands [30], and is regarded as established in Switzerland [26], Austria and Slovenia [29]. Since 2008, *Ae. japonicus* has been continuously recorded in Germany, with potentially three established populations (in the German federal states North Rhine-Westphalia, Baden-Württemberg, Rhineland-Palatinate and Bavaria [18, 29, 53]). Although *Ae. albopictus* has repeatedly been trapped during the last few years in southern Germany (e.g. [22], together with *Ae. japonicus* [54]), it seems questionably if those individuals belong to established populations. In 2015, co-occurrence of *Ae. albopictus* and *Ae. japonicus* was observed in northern Italy [55].

### Temperature and precipitation requirements

In addition to projecting the habitat suitability, we compared the modelled niche functions of the two invasive species by means of one-variable models using MAXENT. As has been suggested by others ([20, 56, 57]), *Ae. albopictus* seems to be adapted to warmer climates than *Ae. japonicus*. Therefore, we expected patterns of habitat suitability in Europe reflecting the species’ temperature requirements (higher temperatures in the south, higher habitat suitability for *Ae. albopictus*; lower temperatures in the north, higher habitat suitability for *Ae. japonicus*)*.* This hypothesis is supported by the one-variable response curves regarding the two temperature variables. The response curves for *Ae. albopictus* show a higher temperature optimum considering the temperatures of coldest and warmest quarter, respectively. *Aedes japonicus* is known to be a cold tolerant mosquito species; however, it has been shown that higher temperature can positively affect the development of larvae of *Ae. japonicus*, at least to some extent, whereas temperatures exceeding a certain temperature may be inhibitory [8]*.* Thus, the southernmost limits of the European range for *Ae. japonicus* might be ascribed to limiting high temperatures.

The optimum of about 25 °C for the mean temperature of the warmest quarter matches the optimal temperature for adult longevity under laboratory conditions and field conditions stated by [58]. Delatte et al. [57] found the optimal temperature for immature stage development to be about 30 °C and is thus slightly above the optimum of 25 °C.

The response curves indicate a narrower temperature niche for *Ae. japonicus* compared to *Ae. albopictus*, which mirrors the formers much smaller native range. The geographically larger native range of *Ae. albopictus* comprises a much wider range of temperatures with higher mean temperatures (Additional file 3: Table S3). It must be kept in mind, however, that the native range niches of species may differ strongly from the invasive range niches (cf. [23]) due to adaptations during the invasion processes.

Considering the modelled precipitation niches the pattern is not as clear. Both species are reliant on the availability of small aquatic habitats for egg deposition, which requires a certain amount of precipitation during summer months. We hypothesized that precipitation requirements would be similar for both species. This assumption is reflected by both species showing quite similar niches considering the variable annual precipitation (i.e. similar requirements in terms of amplitude and optimum of annual precipitation). However, the one variable response curves do not account for interactions between variables. Thus, a certain amount of precipitation in a warmer or cooler climate leads to very different evaporation rates and therefore very different egg laying habitat compositions. It has been suggested that the role of human water supply to provide breeding sites for both species may be even more important than precipitation conditions [49] and it may thus be assumed that there is no clear relationship between the occurrence of *Ae. albopictus* and precipitation. This is in accordance with the comparably low contribution of the precipitation variables to the MAXENT model for *Ae. albopictus*. On the other hand, the relatively high contribution of the variable annual precipitation to the MAXENT model for *Ae. japonicus* which may be explained be the relatively high precipitation amounts within the northern Alpine foothills (Switzerland), where stable populations of *Ae. japonicus* are known (e.g. [19]).

### Interspecific concurrence

*Aedes albopictus* has proven to be a superior competitor to *Ae. japonicus* in artificial container habitats, showing higher overwintering survival and securing more food resources in larval habitats [20, 59]. More specifically, *Ae. albopictus* larvae showed higher survivorship, shorter developmental time and higher estimated population growth rate compared to *Ae. japonicus* in competition experiments [20]*.* This can be partly compensated for by timing of the life-cycle stages of *Ae. japonicus*. Compared to larvae of *Ae. albopictus,* larvae of *Ae. japonicus* can be found earlier in the year [8]. Furthermore, the ability to establish earlier in spring and to remain active for longer in autumn is a characteristic, which, on the one hand, supports the assumed higher cold tolerance of the species, and secondly, allows *Ae. japonicus* to circumvent intense larval competition or avoid temporal overlap of larval stages with those of co-occurring species in the often highly density-dependent, resource-limited environment of the larval habitat [8]. However, compared to *Ae. albopictus, Ae. japonicus* generally seems to have a lower intrinsic capacity for population growth and is considered a weak larval competitor [8].

### Assessment of modelling performance

Two key limitations should be kept in mind when interpreting the results of habitat suitability modelling for *Ae. japonicus*. First, the assumption that the distribution of *Ae. japonicus* in Europe is in equilibrium with its environmental conditions may be violated due to dispersal limitation as the species is considered to be currently in the process of spreading. Secondly, the time period of occurrence data does not match the time period of environmental variables. The occurrence records date from 2009 to 2015; however, empirical data on climatic conditions were not available for Europe for this time period, but only for 1950 up to 2000. Climatic conditions have changed in the interim.

The modelling of *Ae. albopictus* does not pose the same problems as the occurrence records used for modelling also date from 1950 to 2000, thus overlapping with the climate data. Differences in the patterns of projected habitat suitability as well as the modelled niche functions between *Ae. albopictus* and *Ae. japonicus* might not only be attributed to differences in the ecology of the two species but could be ascribed to a certain extent to different assumptions underlying the modelling processes. However, to test whether the same trends still hold true, the incorporation of more recent climatic data would be necessary but was not feasible at the moment.

The use of satellite imagery in species distribution modelling certainly yields valuable information (as recently shown by [60]). However, we here considered climatic effects determine species distribution or habitat suitability at a coarser spatial scale.

### Future distribution

*Aedes albopictus* is considered to be the most invasive mosquito species in the world [6, 24]). Against the background of its vector competence, this species is assumed to become a major threat to public health in Europe [24]. According to our results, there is a clear expansion of the area with modelled habitat suitability for *Ae. albopictus* in Europe under projected climate change. The projected range expansion of *Ae. albopictus* in Europe is in accordance with the assumption that the species is adapted to warmer climatic conditions and will be thus promoted by global warming. In contrast, *Ae. japonicus* is assumed to be adapted to temperate climatic conditions [29]. Under future climatic conditions the potential range of this species (i.e. the area with modelled habitat suitability) seems to decrease. The projected range reduction is probably attributed to increasing temperatures as previously suggested ([8]). Moreover, *Ae. japonicus* is considered a low competing species, and its projected range restriction induced by climate change may additionally be decreased by further spread of *Ae. albopictus* in Europe in the face of climate change [8]. However, the projected decrease of the potential range for *Ae. japonicus* under climate change is based on the assumption that *Ae. japonicus* is not able to adapt to higher temperature [8]. Results from current monitoring suggest that *Ae. japonicus* tends to expand its current European range and will be able to colonise new territories in central Europe [53, 55], facilitated by human-mediated, passive transportation [53].

Both species are reliant on the availability of small aquatic habitats for egg deposition and thus on a certain amount of precipitation ([59]). The projected reduction of precipitation in the face of climate change (IPCC, [61]) is assumed to be less important for the two mosquito species compared to the projected raise in temperature in Europe. However, in the face of climate change, precipitation could become an important limiting factor driving the southernmost borders of the European ranges of these mosquito species.

## Conclusions

Over the last few decades *Aedes albopictus* and *Ae. japonicus* have been accidentally introduced into many countries worldwide, and have shown a rapid and extensive range expansions beyond their native ranges [8]. Both species are assumed to be able to adapt to new climatic conditions outside their native range. Several characteristics of the species (high ecological plasticity, diverse larval habitats and desiccation resistance of eggs; [8]) together with extrinsic factors like increasing tourism and global trade might further promote their invasion success. Due to their vector relevance, further surveillance of the European spread for both species is necessary, focusing on regions where habitat suitability is predicted to be high under future climate scenarios. Although our models for *Ae. albopictus* and *Ae. japonicus* under future climate can be used for predictions, risk assessments and monitoring programmes, we still recommend continuously surveying the establishment and spread of the two vector species and potentially adjust models, e.g. considering the potential of niche evolution of mosquito species.

## Additional files

Additional file 1: Table S1. Spearman correlation coefficients of the six environmental variables provided by worldclim data (www.wordclim.org) within the study area: mean temperature of warmest quarter (bio10); mean temperature of coldest quarter (bio11); temperature range (bio07); precipitation of warmest quarter (bio18); annual precipitation (bio12); precipitation seasonality (bio15). (DOCX 14 kb)
Additional file 2: Table S2. AUC values for the single models as well as for the consensus model resulting from the Ensemble forecasting including all ten single models. (DOCX 13 kb)
Additional file 3: Table S3. Temperature conditions for *Aedes albopictus* and *Ae. japonicus* within the native range derived from the worldclim data (www.wordclim.org) (DOCX 15 kb)
Additional file 4: Figure S1. Observed European distribution for *Aedes albopictus* and *Ae. japonicus*. Occurrence records as used for modelling: assembled by Kraemer MUG et al. [25] and Koch et al. [17] for *Ae. albopictus* and by Schaffner et al. [26]; Huber et al. [27]; Huber et al. [19]; Krebs et al. [28]; Zielke et al. [29]; Melaun et al. [18] and Zielke et al. [30] for *Ae. japonicus*. The records were adjusted to the raster of the environmental variables (about 10 × 10 km). (PNG 5199 kb)

## Abbreviations

ANN: Artificial neural networks
AUC: Area under the receiver operating characteristic curve
CTA: Classification tree analysis
FDA: Flexible discriminant analysis
GAM: Generalized additive models
GBM: Generalized boosted models
GLM: Generalized linear models
IPCC: Intergovernmental Panel on Climate Change
MARS: Multivariate adaptive regression splines
MAXENT: Maximum entropy approach
RF: Random forest algorithm
SDM: Species distribution model
SRE: Surface range envelope
