# Erratum to: *Aedes albopictus* and *Aedes japonicus* - two invasive mosquito species with different temperature niches in Europe

**DOI:** 10.1186/s13071-016-1893-7

**Published:** 2016-12-06

**Authors:** Sarah Cunze, Lisa K. Koch, Judith Kochmann, Sven Klimpel

**Affiliations:** 1 Institute of Ecology, Evolution and Diversity, Goethe-University, D-60438 Frankfurt/ M, Germany; 2 Senckenberg Biodiversity and Climate Research Centre, Senckenberg Gesellschaft für Naturforschung, D-60438 Frankfurt/ M, Germany

## Erratum

After the publication of our paper [1] we realised that, due to erroneous labelling of some figure files, Figs. 3, 4 and 5 appear out of order and are associated with wrong figure legends. The corrected versions of the three figures are included below.

**Fig. 3:**
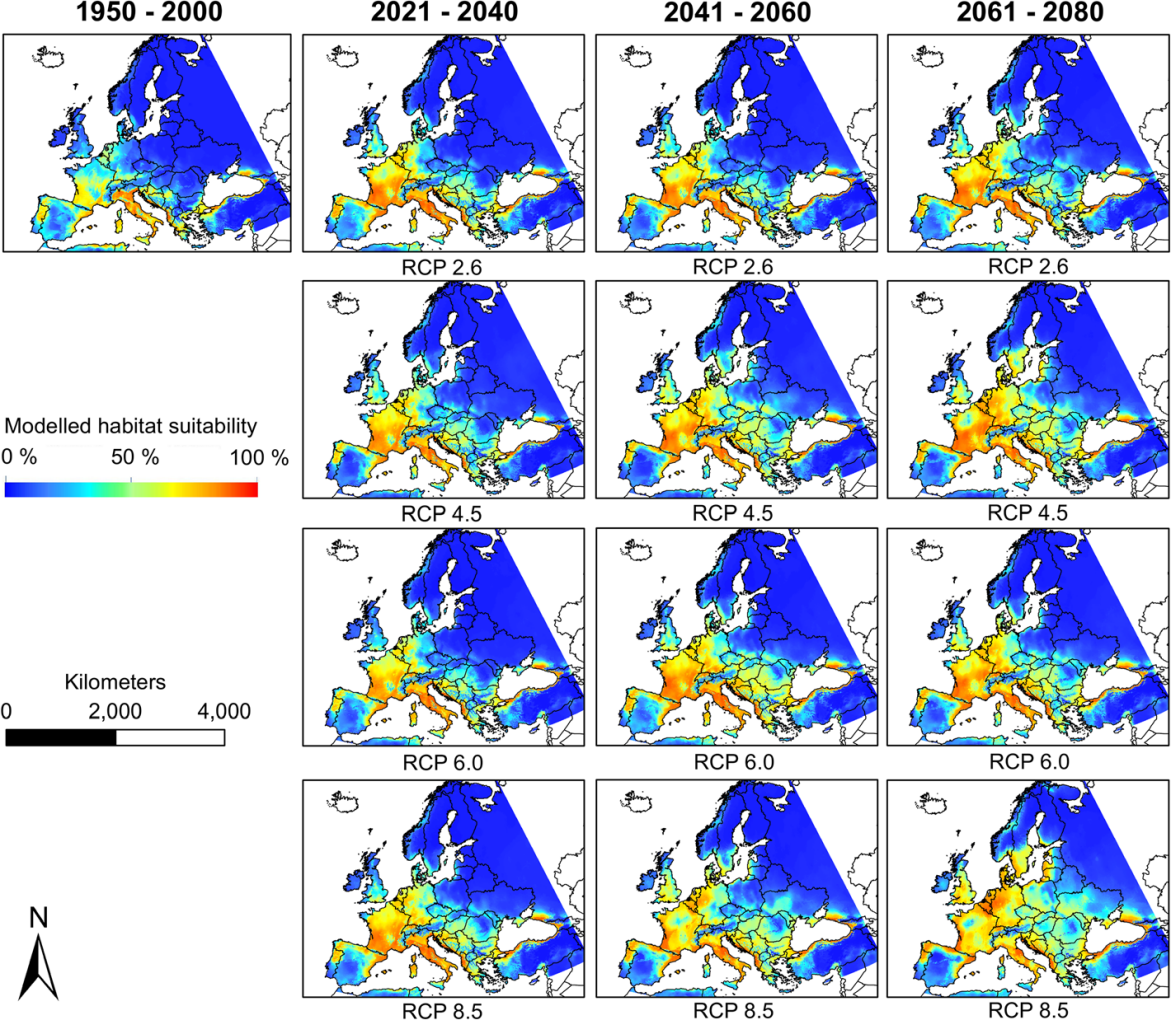
Modelled habitat suitability (Ensemble forecasting) for *Aedes albopictus* under current and future climatic conditions

**Fig. 4:**
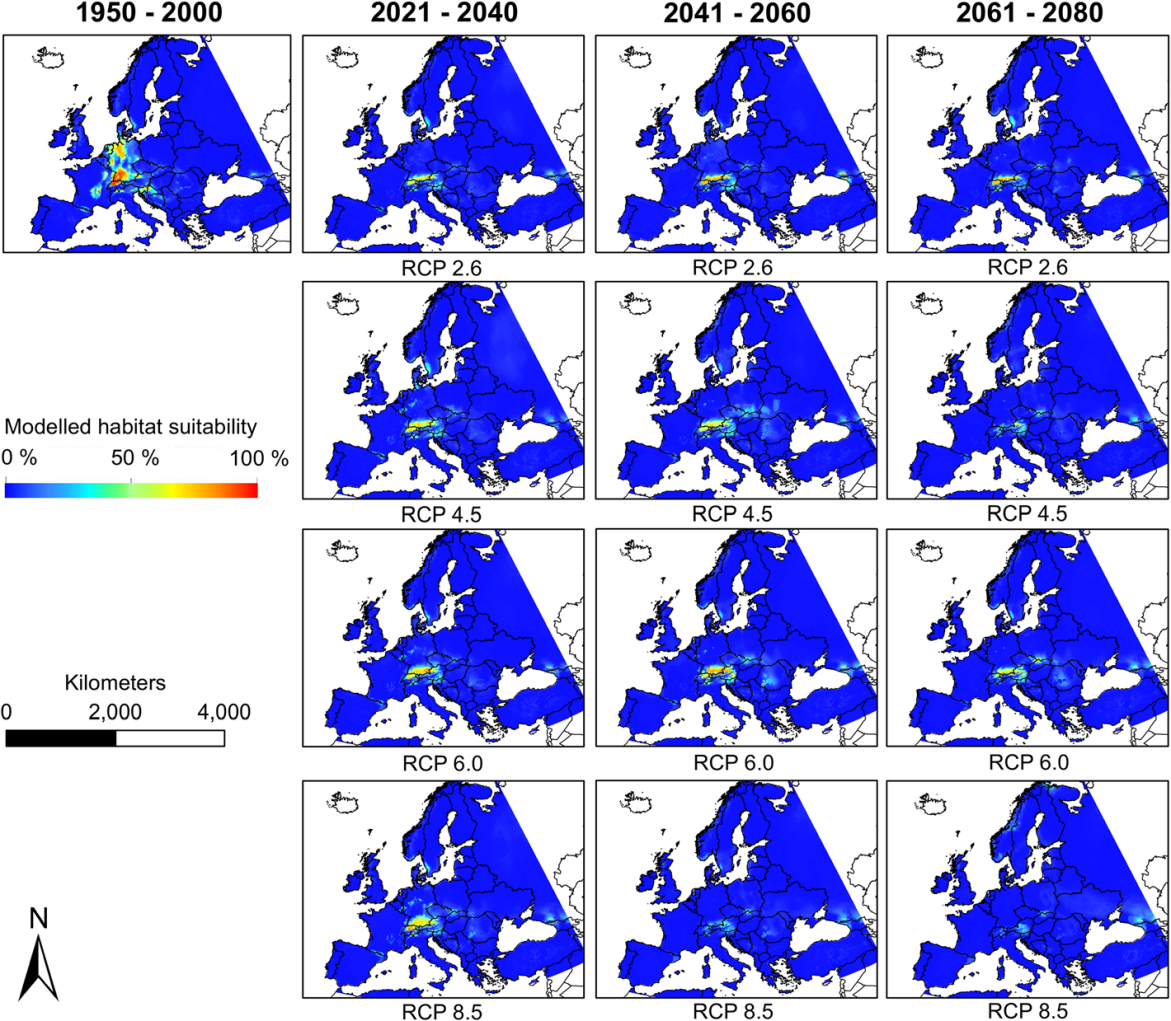
Modelled habitat suitability (Ensemble forecasting) for *Aedes japonicus* under current and future climatic conditions

**Fig. 5:**
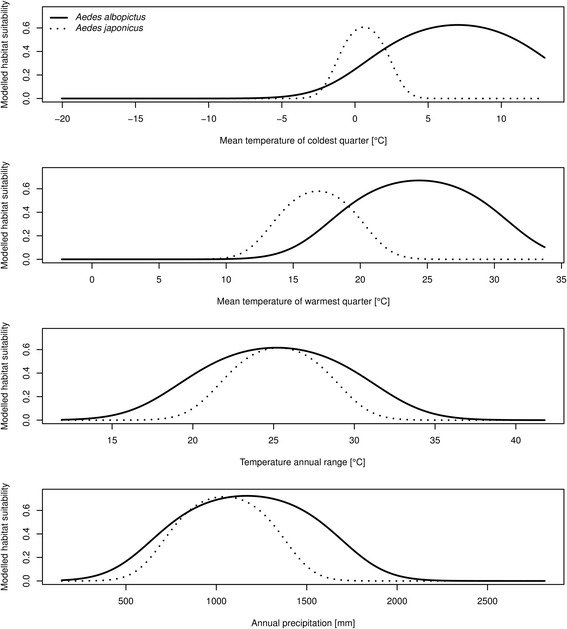
One-variable-response-curves for *Aedes albopictus* (*solid line*) and *Aedes japonicus* (*dotted line*) considering the different environmental variables (required: AUC value for the one variable model for both species > 0.75, see Table 2)

We would like to apologise for this error and for any inconvenience this may have caused.
