# Supplementary material for: Aedes albopictus and Aedes japonicus - two invasive mosquito species with different temperature niches in Europe
Source: Parasit Vectors. 2016 Nov 4;9:573. doi: 10.1186/s13071-016-1853-2 (PMC5097377; doi:10.1186/s13071-016-1853-2)
Supplement: Additional file 1: Table S1. — Spearman correlation coefficients of the six environmental variables provided by worldclim data (www.wordclim.org) within the study area: mean temperature of warmest quarter (bio10); mean temperature of coldest quarter (bio11); temperature range (bio07); precipitation of warmest quarter (bio18); annual precipitation (bio12); precipitation seasonality (bio15). (DOCX 14 kb) [file 13071_2016_1853_MOESM1_ESM.docx]

Table S1: Spearman correlation coefficients of the six environmental variables provided by worldclim data (www.wordclim.org) within the study area: mean temperature of warmest quarter – bio10; mean temperature of coldest quarter – bio11; temperature range – bio07; precipitation of warmest quarter – bio18; annual precipitation – bio12; precipitation seasonality – bio15

|  | **bio10** | **bio11** | **bio07** | **bio18** | **bio12** | **bio15** |
| --- | --- | --- | --- | --- | --- | --- |
| **bio10** | 1 | 0.67 | 0.08 | -0.49 | -0.19 | 0.13 |
| **bio11** | 0.67 | 1 | -0.61 | -0.31 | 0.27 | -0.06 |
| **bio07** | 0.08 | -0.61 | 1 | -0.12 | -0.54 | 0.32 |
| **bio18** | -0.49 | -0.31 | -0.12 | 1 | 0.57 | -0.17 |
| **bio12** | -0.19 | 0.27 | -0.54 | 0.57 | 1 | -0.27 |
| **bio15** | 0.13 | -0.06 | 0.32 | -0.17 | -0.27 | 1 |
