# Supplementary material for: Aedes albopictus and Aedes japonicus - two invasive mosquito species with different temperature niches in Europe
Source: Parasit Vectors. 2016 Nov 4;9:573. doi: 10.1186/s13071-016-1853-2 (PMC5097377; doi:10.1186/s13071-016-1853-2)
Supplement: Additional file 2: Table S2. — AUC values for the single models as well as for the consensus model resulting from the Ensemble forecasting including all ten single models. (DOCX 13 kb) [file 13071_2016_1853_MOESM2_ESM.docx]

Table S2: AUC values for the single models as well as for the consensus model resulting from the Ensemble forecasting including all ten single models.

|  | ***Ae. albopictus*** | ***Ae. japonicus*** |
| --- | --- | --- |
| **GLM** | 0.942 | 0.995 |
| **GAM** | 0.964 | 0.944 |
| **GBM** | 0.955 | 0.997 |
| **CTA** | 0.944 | 0.986 |
| **ANN** | 0.942 | 0.992 |
| **SRE** | 0.819 | 0.898 |
| **FDA** | 0.936 | 0.986 |
| **MARS** | 0.948 | 0.995 |
| **RF** | 1.00 | 1.00 |
| **MAXENT** | 0.950 | 0.995 |
| **Consensus model** | 0.972 | 0.999 |
