# Supplementary material for: Aedes albopictus and Aedes japonicus - two invasive mosquito species with different temperature niches in Europe
Source: Parasit Vectors. 2016 Nov 4;9:573. doi: 10.1186/s13071-016-1853-2 (PMC5097377; doi:10.1186/s13071-016-1853-2)
Supplement: Additional file 3: Table S3. — Temperature conditions for Aedes albopictus and Ae. japonicus within the native range derived from the worldclim data (www.wordclim.org) (DOCX 15 kb) [file 13071_2016_1853_MOESM3_ESM.docx]

Table S3: Temperature conditions for *Aedes albopictus* and *Ae. japonicus* within the native range derived from the worldclim data (www.wordclim.org)

|  | *Ae. albopictus* | *Ae. japonicus* |
| --- | --- | --- |
| a) mean temperature of the warmest quarter [°C] |  |  |
| min | -1.10 | 11.80 |
| max | 35.00 | 28.40 |
| mean | 21.54 | 21.30 |
| std | 7.60 | 3.13 |
|  |  |  |
| b) mean temperature of the coldest quarter [°C] |  |  |
| min | -31.40 | -19.20 |
| max | 27.30 | 19.00 |
| mean | 1.971 | -1.89 |
| std | 16.56 | 6.12 |
