# Supplementary figures and images for: Aedes albopictus and Aedes japonicus - two invasive mosquito species with different temperature niches in Europe
Source: Parasit Vectors. 2016 Nov 4;9:573. doi: 10.1186/s13071-016-1853-2 (PMC5097377; doi:10.1186/s13071-016-1853-2)

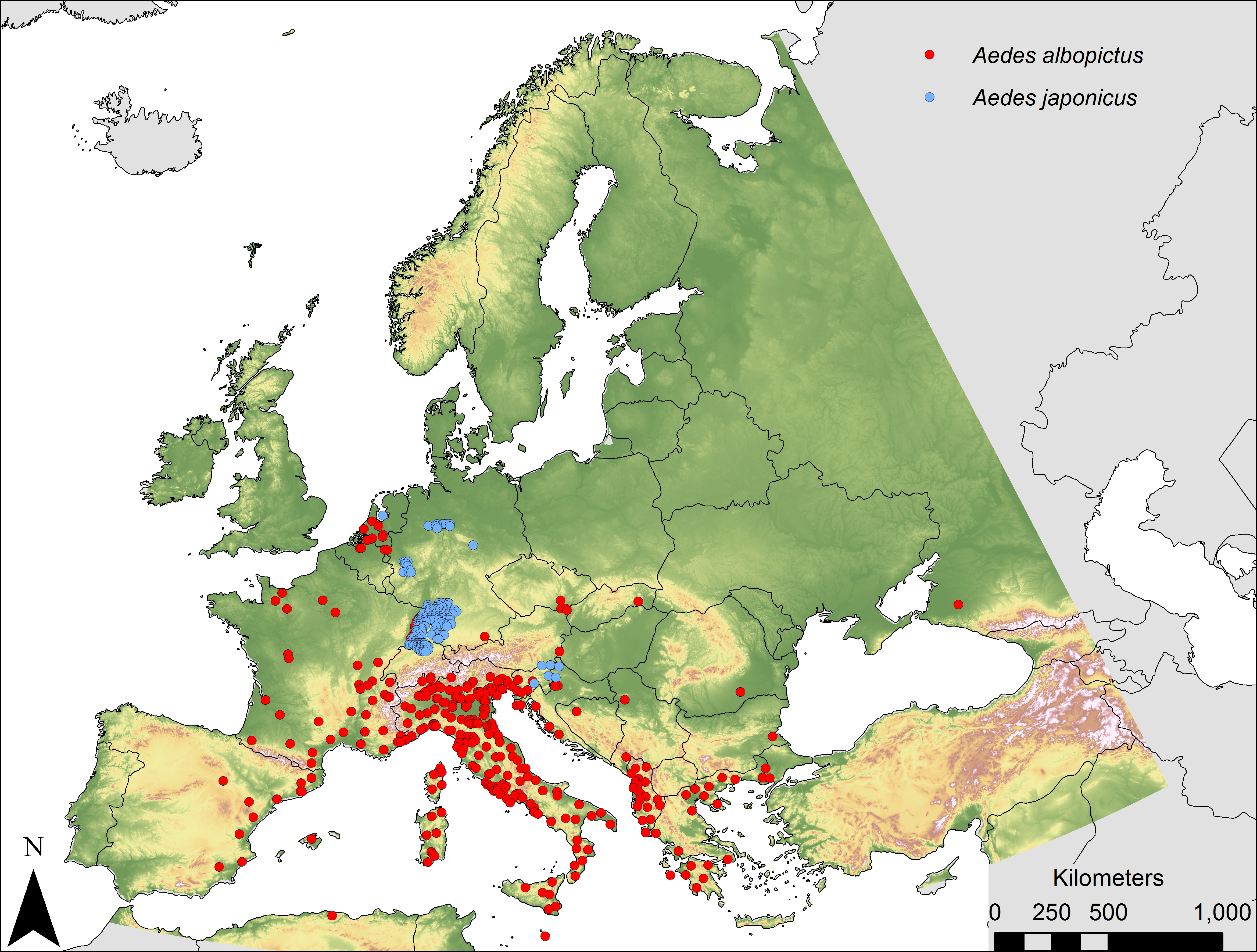

Supplement: Additional file 4: Figure S1. — Observed European distribution for Aedes albopictus and Ae. japonicus. Occurrence records as used for modelling: assembled by Kraemer MUG et al. [25] and Koch et al. [17] for Ae. albopictus and by Schaffner et al. [26]; Huber et al. [27]; Huber et al. [19]; Krebs et al. [28]; Zielke et al. [29]; Melaun et al. [18] and Zielke et al. [30] for Ae. japonicus. The records were adjusted to the raster of the environmental variables (about 10 × 10 km). (PNG 5199 kb) [file 13071_2016_1853_MOESM4_ESM.png]
